# Adipokines and Vascular Endothelial Growth Factor in Normal Human Breast Tissue in Vivo – Correlations and Attenuation by Dietary Flaxseed

**DOI:** 10.1007/s10911-016-9352-9

**Published:** 2016-04-08

**Authors:** Vivian Morad, Annelie Abrahamsson, Preben Kjölhede, Charlotta Dabrosin

**Affiliations:** Linköping University, Linköping, Sweden

**Keywords:** Flaxseed, Diet, Microdialysis, Estrogen, Leptin, Adiponectin

## Abstract

Exposure to sex steroids increases the risk of breast cancer but the exact mechanisms are yet to be elucidated. Events in the microenvironment are important for carcinogenesis. Diet containing phytoestrogens can affect the breast microenvironment and alter the risk of breast cancer. It has previously been shown that estrogen regulates extracellular levels of leptin, adiponectin, and VEGF in normal breast tissue in vivo*.* Whether these proteins correlate in breast tissue in vivo or if diet addition of flaxseed, a major source of phytoestrogens in Western diets, alters adipokine levels in breast tissue are unknown. We used microdialysis to sample proteins of normal human breast tissue and abdominal subcutaneous fat in situ in 34 pre-and postmenopausal women. *In vitro*, co-culture of breast cancer cells and primary human adipocytes was used. *In vivo*, in normal breast tissue, a significant positive correlation between VEGF and leptin was detected. No correlations were found in fat tissue. Co-culture of adipocytes and breast cancer cells *per se* increased the secretion of VEGF and leptin and enhanced the effects of estradiol compared to culture of either cell type alone. *In vitro*, inhibition of VEGF diminished the release of leptin while inhibition of leptin had no influence on VEGF secretion. The levels of leptin decreased and adiponectin increased after a dietary addition of 25 g of flaxseed/day for one menstrual cycle. We conclude that VEGF and leptin correlate significantly in normal human breast tissue *in vivo* and that dietary addition of flaxseed affect adipokine levels in the breast.

## Introduction

The incidence of breast cancer is increasing in the Western world. In breast cancer prevention studies, anti-estrogen therapies reduced the incidence of breast cancer by approximately 50 %, but these therapies are associated with severe side effects such as endometrial cancer, osteoporosis, impaired quality of life, and thromboembolism [1, 2]. Although sex steroids, including estrogens, have been shown to affect breast cancer risk by receptor-mediated processes or via receptor independent mechanisms such as generation of genotoxic metabolites, further mechanisms behind sex steroid induced breast cancer need to be elucidated [3, 4]. As anti-estrogen drugs are associated with poor compliance to the therapy, breast cancer preventive strategies associated with less side-effects, such as diet modifications need to be developed. The tissue microenvironment is recognized as a critical player in all steps of carcinogenesis including breast cancer progression [5]. Increased knowledge of the regulation of the breast tissue microenvironment it is therefore essential for the development of novel breast cancer prevention interventions.

Adipocytes are one of the most abundant cell types in the breast microenvironment, which is characterized by the close interaction between the different cells types in the breast. Adipocytes actively secrete proteins including adipokines that play an important role in the cell-cell interactions in the breast [6]. The adipokine leptin and its receptor may be found in both normal and malignant mammary tissue [7]. In breast cancer, leptin has been associated with increased angiogenesis and vascular endothelial growth factor (VEGF) one of the most potent stimulators of angiogenesis [8, 9]. Up-regulation of intratumoral and/or circulating VEGF has been associated with poor prognosis in many solid tumors including breast cancer [10]. Another adipokine, adiponectin, has been shown to be a key player in several physiological processes and low levels of circulating adiponectin have been associated with breast cancer [11–13]. As adiponectin may counteract the effects of leptin, the ratio of leptin:adiponectin, has been suggested to be an improved indicator of the biological outcome rather than their individual concentrations [14]. It has previously been shown that estradiol plays an important role in the regulation of the extracellular levels of leptin, adiponectin, and VEGF in normal human breast *in vivo* [15–20]. How or if leptin, adiponectin, and/or VEGF correlate or regulate each other in normal human breast tissue *in vivo* is to date unknown. Previous data has suggested that a dietary addition of 25 g of flaxseed/day to premenopausal women affected several inflammatory and angiogenesis regulators in the extracellular environment in normal human breast tissue *in vivo* [15, 21]. It is not known if flaxseed affects adipokine levels in the breast. Our aims of this study were to investigate a possible relationship of the *in vivo* levels of leptin, adiponectin, and VEGF in normal human breast tissue in pre- and postmenopausal women and to explore their reciprocal regulation in an in vitro co-culture model. Moreover, we investigated if a daily addition of flaxseed to the diet for one month altered the adipokine levels in normal human breast tissue in premenopausal women.

## Materials and Methods

### Human Subjects and Experimental Procedure

The regional ethical review board of Linköping approved the study and all participants gave their informed consent. As it previously has been shown that several angiogenesis factors and adipokines are influences by estrogen levels [15, 20–22] women during various stages of the menstrual cycle as well as postmenopausal women with low estrogen levels were included in order to take the hormonal fluctuations into account. A total of 34 women were included in the microdialysis investigations in three different cohorts;

1. Thirteen premenopausal healthy volunteers (age 20–30 years) with a history of regular menstrual cycles (27–35 days) were investigated with microdialysis both in breast tissue and abdominal subcutaneous (s.c.) fat. Nine of them were investigated in both the follicular and luteal phases of one menstrual cycle whereas four premenopausal women were investigated in the luteal phase of one menstrual cycle.
2. Eleven postmenopausal women (age 58–78) that previously had undergone surgery for early breast cancer were investigated with microdialysis in their normal unaffected breast and in abdominal s.c. fat except for two women where the fat microdialysis catheter was omitted due to technical issues.
3. For the diet study another 10 premenopausal women were investigated with microdialysis in normal breast tissue and abdominal s.c. fat tissue in the luteal phase of a menstrual cycle before the start of the diet addition with flaxseed (un-exposed). Thereafter they added 25 g of freshly ground flaxseed/day to their regular diet and a second microdialysis investigation took place in the luteal phase of the next menstrual cycle (exposed). The women were monitored using luteinizing hormone (LH) in urine samples. After the LH peak the first microdialysis investigation was performed within 5–10 days. The next microdialysis investigation was performed at the same day after the LH-peak as in the first un-exposed cycle i.e. if the first microdialysis was performed at day 5 after the LH-peak the next microdialysis investigation was also performed at day 5. All women had been off sex steroid containing medication such as contraceptive pills or hormone replacement therapy for more than three months.

Before the microdialysis catheter insertions, 0.5 ml lidocaine (10 mg/ml) was injected intracutaneously. One microdialysis catheter was placed in the upper lateral quadrant of the left breast in the premenopausal women or in upper lateral quadrant of the unaffected breast in the postmenopausal women as previously described [16, 23–25]. Microdialysis catheters (CMA 71/Microdialysis AB, Solna, Sweden), with a tubular dialysis membrane (100 kDa cutoff, 20 mm membrane length), were used. The catheters were connected to a pump (CMA 107, CMA/Microdialysis AB) and perfused with NaCl 154 mM and hydroxyethyl starch 60 g/l (Voluven®, Fresenius Kabi, Uppsala, Sweden) at 0.5 μl/min. After a 60 min equilibration period the sample were collected and stored at −70 °C. All microdialysis values are given as original raw data.

For the *in vitro* tissue culture study, abdominal s.c. fat tissue was obtained during elective surgery. These women were not included in the microdialysis investigations.

### Cell Culture

MCF7 cells (HTB-22; Human breast adenocarcinoma, estrogen receptor (ER) and progesterone receptor positive), obtained from the American type culture collection (ATCC; Manassas, Va, USA), were maintained in DMEM without phenol red (Invitrogen, Carlsbad, CA, USA) supplemented with 10 % fetal bovine serum (FBS) (Invitrogen), 2 mM glutamine (Invitrogen) 50 IU/ml penicillin-G and 50 μg/ml streptomycin (Invitrogen), at 37 °C in a humidified atmosphere of 5 % CO_2_. Confluent MCF7 cells were trypsinized and seeded in 6 wells plates (Falcon 3046), 25,000 cells/cm^2^, for 24 h at 37°C in a humidified atmosphere of 5 % CO_2_ before starting the co-culture.

### Isolation of Adipocytes

Collagenase digestion (type 1, Worthington, NJ, USA) in modified Krebs-Ringer solution (0.13 M NaC1, 5 mM KCl, 2.5 mM CaC1_2_, 1.2 mM MgSO_4_, 0.5 mM inorganic phosphate, 25 mM Hepes, pH 7.4, 1 % bovine serum albumin, 200 nM adenosine, 2 mM glucose), were used to isolate mature adipocytes at 37 °C.

### Hormone Treatments of Co-Cultured Adipocytes and MCF7 Cells *In Vitro*

A fixed volume of isolated mature adipocytes, diluted in an equal volume of modified Krebs-Ringer solution and double volume of DMEM containing 7 % bovine serum albumin, 200 nM PIA, 250 nM Hepes, were added to pre-seeded MCF7 cells in 6 well plates. The co-culture or each cell type alone were cultured in serum-free medium consisting of a 1:1 mixture of nutrient mixture F-12 (GIBCO, Paisley, UK) and Dulbecco’s modified Eagle’s medium without phenol red (GIBCO, Paisley, UK) supplemented with transferrin (10 μg/ml; Sigma), insulin (1 μg/ml; Sigma), and bovine serum albumin (0.2 mg/ml; Sigma) with or without 10^−9^ M estradiol, or the combination of 10^−9^ M estradiol and 10^−6^ M fulvestrant. Adipocytes from one single volunteer were used for each experimental set-up and repeated with fat from another donor. For the inhibition assays co-cultures were treated with anti-human leptin antibody (Genway, USA), anti-human VEGF antibody (Calbiochem, USA), or normal IgG from the same species (R&D system, USA) at 0.1 μg/ml and 1 μg/ml in presence or absence of 10^−9^ M estradiol. After 48 h treatment, the condition medium was collected and stored at −70 °C for subsequent analysis.

### Quantification of Leptin, Adiponectin, and VEGF

The human immunoassay kits (R&D systems, Minneapolis, USA) were used for leptin (intra-assay variation of 3 % and inter-assay variation of 4 %), adiponectin (intra-assay variation of 4 % and inter-assay variation of 7 %), and VEGF (intra-assay variation of 5 % and inter-assay variation of 7 %) detection in culture media and microdialysates.

### Control of Efficacy of Treatments

The efficacy of the antibodies was confirmed by measuring the concentrations of the protein in the co-cultures with and without specific antibodies; leptin levels decreased from 8800 ± 40 pg/ml with control antibody compared to 12 ± 0.0 pg/ml with anti-leptin antibody (*n* = 5) and VEGF levels decreased from 533 ± 16 pg/ml in presence of control antibody compared to 3 ± 0.01 pg/ml with anti-VEGF antibody (*n* = 5). Fulvestrant is a registered anti-estrogen drug (Faslodex®) and its effect was confirmed in our laboratory by measuring proliferation of hormone dependent breast cancer cells using 5-bromo-2′-deoxyuridine (BrdU) proliferation kit according to the manufacturer’s guidelines (BrdU cell proliferation ELISA, Roche, Mannheim, Germany). Fulvestrant potently reversed the estradiol-induced proliferation of hormone dependent breast cancer cells; relative proliferation in control cells 1 ± 0.02, in E2 exposed cells 4 ± 0.2 and in E2 + fulvestrant exposed cells 0.8 ± 0.02 (*n* = 8 in each group).

### Statistical Analyses

Statistical analyses were performed using GraphPad Prism software 6.0. Pearson’s correlation coefficient, Wilcoxon signed rank test, or student’s t test were used as appropriate. Data are expressed as mean ± SEM. A *p* < 0.05 was considered as statically significant.

## Results

### VEGF Correlated significantly to Leptin in Normal Human Breast Tissue

Microdialysis was performed in nine premenopausal women in the follicular and luteal phases of one menstrual cycle (18 occasions), in another 14 premenopausal women in one luteal phase (14 occasions), and in 11 postmenopausal women (11 occasions), which equals 43 occasions. During each occasion one microdialysis catheter was inserted in normal breast tissue and another catheter was inserted in abdominal s.c. fat except for two occasions where the microdialysis of the abdominal s.c. fat was omitted. Thus, 43 microdialysis investigations were performed in breast tissue and 41 microdialysis investigations were performed in abdominal s.c. fat. There were no subsequent complications after the microdialysis investigations.

**Fig. 1:**
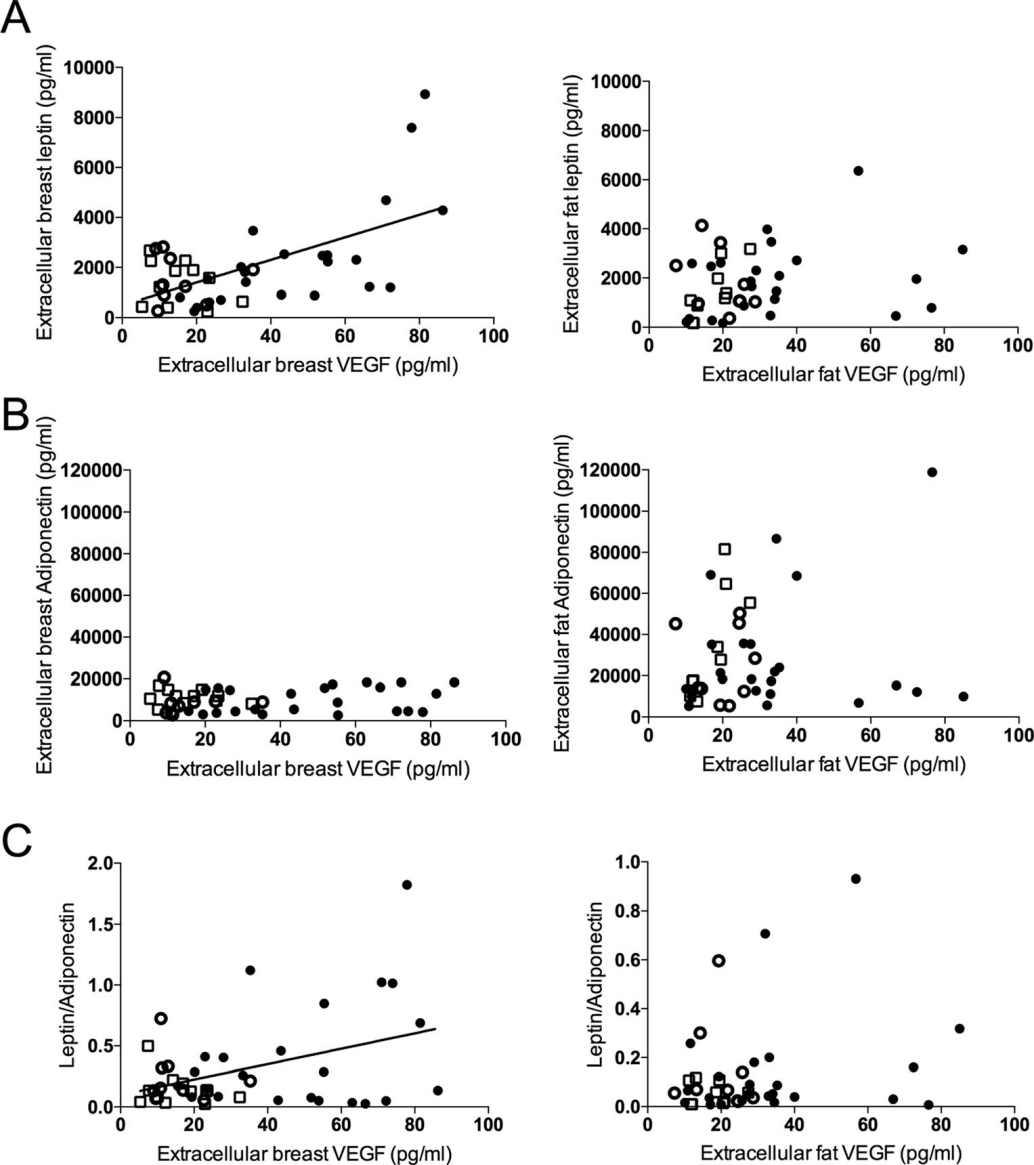
*VEGF correlated with leptin and the ratio of leptin*:*adiponectin in normal human breast tissue.* Microdialysis was used for sampling of extracellular VEGF, leptin, and adiponectin in vivo in normal breast tissue (extracellular breast) and the abdominal subcutaneous (s.c.) fat (extracellular fat), in pre-and postmenopausal women. Filled circles represent women investigated in the luteal phase of the menstrual cycle and open circles women investigated in the follicular phase of the menstrual cycle. Open squares represent postmenopausal women. **a**. Extracellular VEGF levels correlated positively with extracellular leptin levels in normal breast tissue, *r* = 0.6; *p* < 0.0001; *n* = 43, but not in the abdominal s.c. fat, *r* = 0.22; *p* = 0.15; *n* = 41. **b**. VEGF did not correlate with adiponectin in normal breast tissue, *r* = 0.15; *p* = 0.32; *n* = 43, or abdominal s.c. fat tissue, *r* = 0.16; *p* = 0.31; *n* = 41. **c**. There was a significant positive correlation between VEGF and the ratio of leptin to adiponectin in normal breast tissue, *r* = 0.4; *p* < 0.01; *n* = 43, whereas no correlation was found in abdominal s.c. fat tissue, *r* = 0.23; *p* = 0.14; *n* = 41

There was a significant positive correlation between extracellular VEGF levels and extracellular leptin levels in breast tissue, *r* = 0.6; *p* < 0.001; *n* = 43, but not in the abdominal s.c. fat, Fig. 1a.

No significant correlations were found between VEGF and adiponectin in either tissue, Fig. 1b. A significant positive correlation was found between VEGF and leptin:adiponectin ratio in the breast, *r* = 0.4; *p* < 0.01; *n* = 43, but not in abdominal s.c. fat, Fig. 1c.

Extracellular breast and extracellular fat VEGF correlated significantly (*r* = 0.49) as well as extracellular breast and extracellular leptin levels (*r* = 0.50). No correlation was found between extracellular breast and extracellular fat adiponectin (*r* = 0.08).

### VEGF Inhibition Decreased Leptin Levels but not Vice Versa

To be able to explore if the release of leptin was dependent on VEGF or vice versa, we set up *in vitro* cell culture experiments. As we also wanted to explore possible effects of estradiol we co-cultured freshly isolated human adipocytes with the ER+ MCF7 breast cancer cells since established normal human breast epithelial cell lines lack the expression of ER [26, 27].

**Fig. 2:**
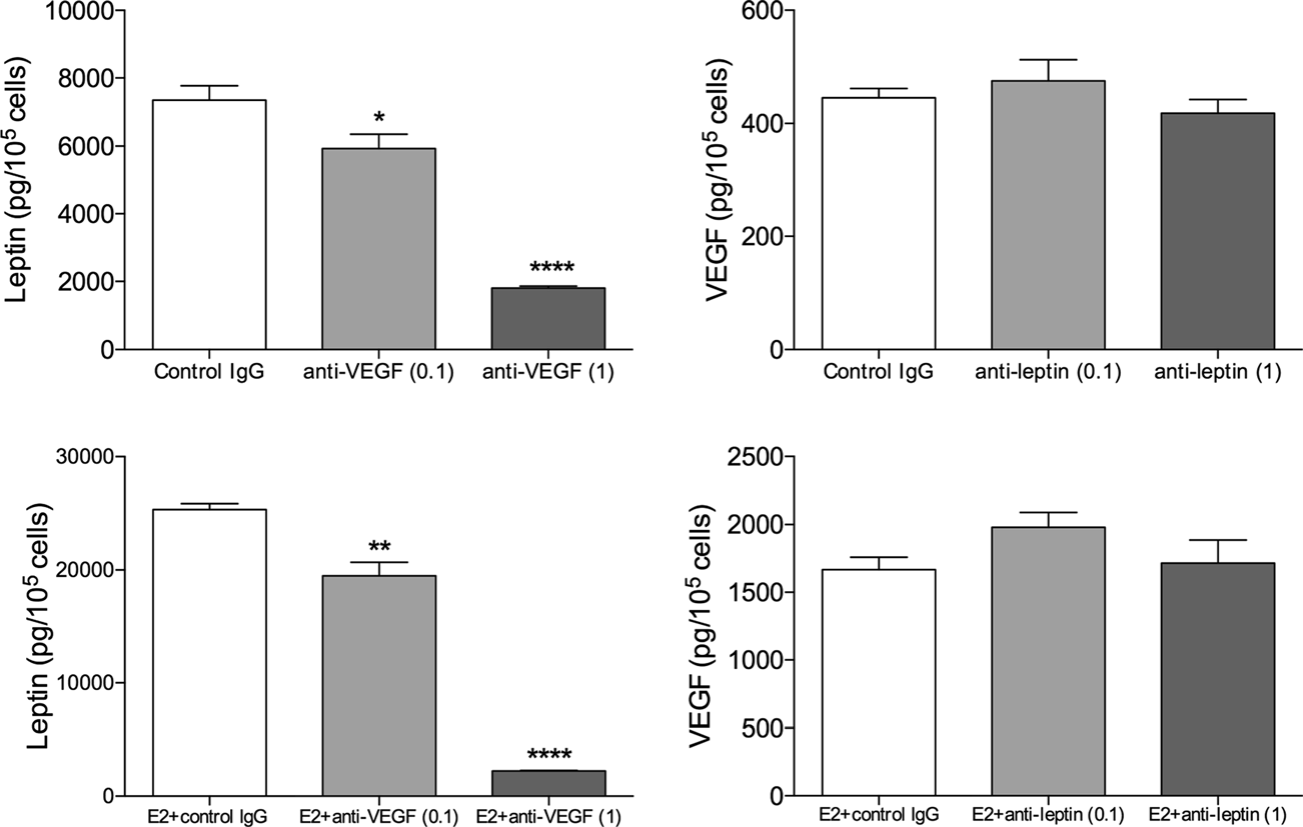
*Inhibition of VEGF decreased leptin secretion in co-culture of MCF7 and adipocytes.* Co-culture of ER+ breast cancer cells, MCF7, and freshly isolated human adipocytes, were exposed to; **a**. Anti-VEGF antibody at 0.1 μg/ml and 1 μg/ml. Extracellular levels of leptin were analyzed after 48 h. *, *p* < 0.05 and ****, *p* < 0.0001 compared with controls, *n* = 5–6 in each group. **b**. Anti-leptin antibody at 0.1 μg/ml and 1 μg/ml. Extracellular levels of VEGF were analyzed after 48 h. *n* = 5–6 in each group. **c**. Anti-VEGF antibody at 0.1 μg/ml and 1 μg/ml in the presence of estradiol (10^−9^ M). Extracellular levels of leptin were analyzed after 48 h. ***, *p* < 0.001 and ****, *p* < 0.0001 compared with controls, *n* = 5–6 in each group. **d**. Anti-leptin antibody at 0.1 μg/ml and 1 μg/ml in presence of estradiol (10^−9^ M). Extracellular levels of VEGF were analyzed after 48 h. *n* = 5–6 in each group

In co-cultures, treatment with an anti-VEGF antibody resulted in a dose-dependent inhibition of extracellular leptin levels whereas inhibition of leptin did not alter the release of VEGF, Fig. 2.

Next, we investigated if inhibition of the different proteins with antibody treatments to co-ltures was effective even in presence of estradiol. As shown in, Fig. 2c-d, inhibition of VEGF resulted in a dose-dependent decrease of leptin levels even in presence of estradiol whereas inhibition of leptin did not result in any change of VEGF levels.

### Co-Culture with Adipocytes Enhanced the Effects of Estradiol on VEGF and Leptin Secretion

**Fig. 3:**
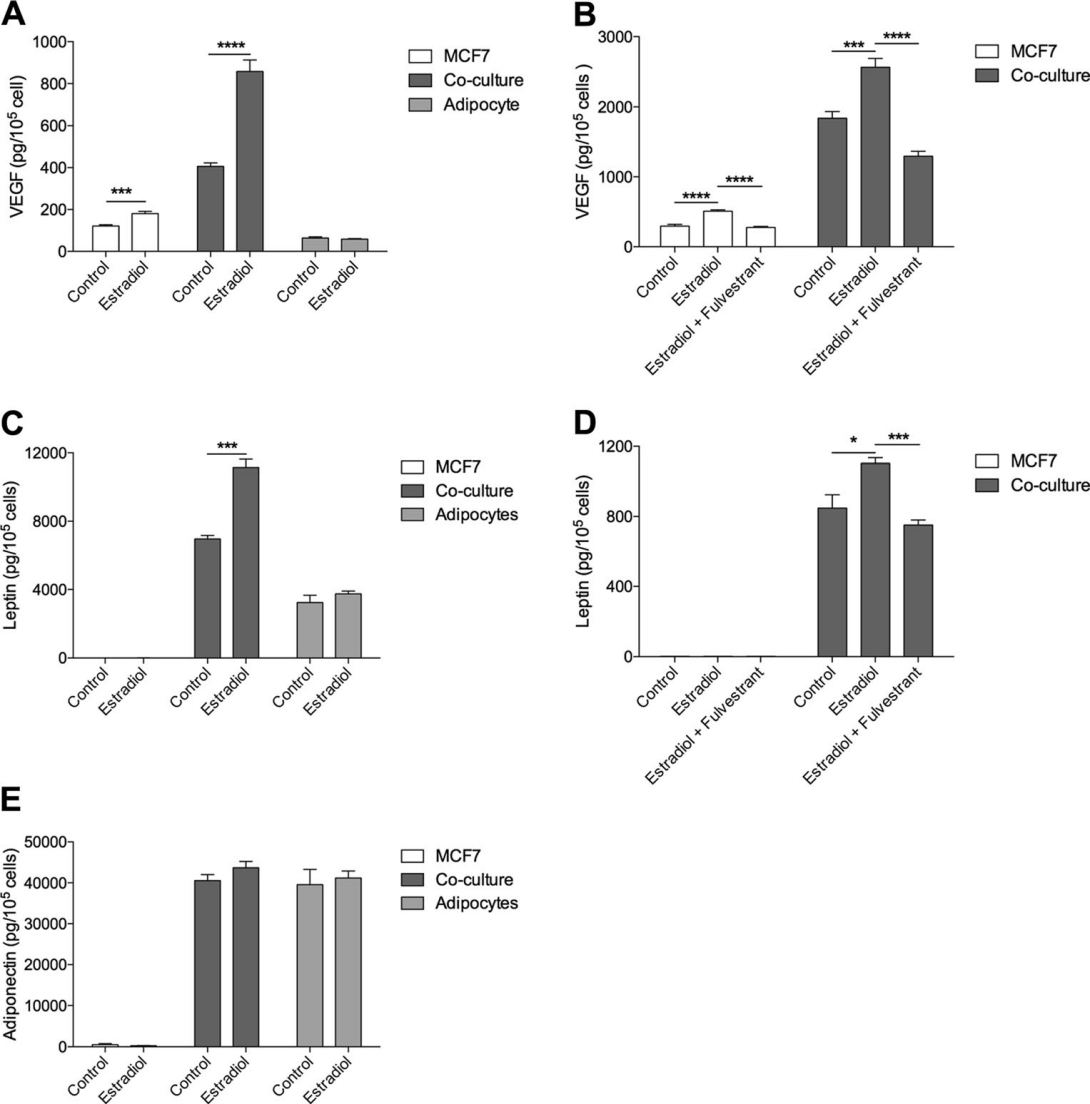
Co-culture with adipocytes enhanced the effects of estradiol on leptin and VEGF secretion. A. MCF7 cells, freshly isolated human adipocytes or co-culture of MCF7 and adipocytes, were cultured with or without estradiol (10^−9^ M) for 48 h. Extracellular levels of VEGF were measured in the conditioned medium with ELISA. ***, *p* < 0.001 and ****, *p* < 0.0001, *n* = 6. B. MCF7 cells alone or co-cultured with adipocytes were treated with estradiol (10^−9^ M) or estradiol (10^−9^ M) and fulvestrand (10^−6^ M) for 48 h. Extracellular levels of VEGF were measured with ELISA in the conditioned medium. ***, *p* < 0.001 and ****, *p* < 0.0001, *n* = 6. C. MCF7 cells, adipocytes or co-culture of MCF7 and adipocytes, were cultured with or without estradiol (10^−9^ M) for 48 h. Extracellular levels of leptin were measured with ELISA in the conditioned medium. ***, *p* < 0.001, *n* = 6. D. MCF7 cells alone or co-cultured with adipocytes were treated with estradiol (10^−9^ M) or estradiol (10^−9^ M) and fulvestrant (10^−6^ M) for 48 h. Extracellular levels of leptin were measured with ELISA in the conditioned medium. *, *p* < 0.05 and ***, *p* < 0.001, *n* = 6. E. MCF7 cells, adipocytes or co-culture of MCF7 and adipocytes, were cultured with or without estradiol (10^−9^ M) for 48 h. Extracellular levels of adiponctin were measured with ELISA in the conditioned medium. *n* = 6 in each group

Co-culture *per se* increased the extracellular levels of VEGF and leptin compared with the different cell types cultured alone, Fig. 3a and c.

The release of VEGF and leptin from adipocytes was unaffected by estradiol treatment, Fig. 3a and c. However, estradiol exposure significantly increased the extracellular levels of VEGF and leptin from co-cultures to a much higher degree than in MCF7 cells alone, Fig. 3a and c. In the case of leptin, MCF7 cells cultured alone did not secrete any detectable levels whereas in co-culture the levels were more than doubled in the control group without hormone treatment compared with adipocytes alone, and tripled after estradiol exposure, Fig. 3c.

The pure anti-estrogen fulvestrant reversed the increase of VEGF and leptin after estradiol exposure confirming the role of the ER in the regulation of these proteins, Fig. 3b and d.

Adiponectin levels were unaltered in adipocytes compared with co-cultures with or without estradiol treatment, Fig. 3e.

### Dietary Addition of Flaxseed Decreased Leptin Levels and Increased Adiponectin in Breast Tissue

**Fig. 4:**
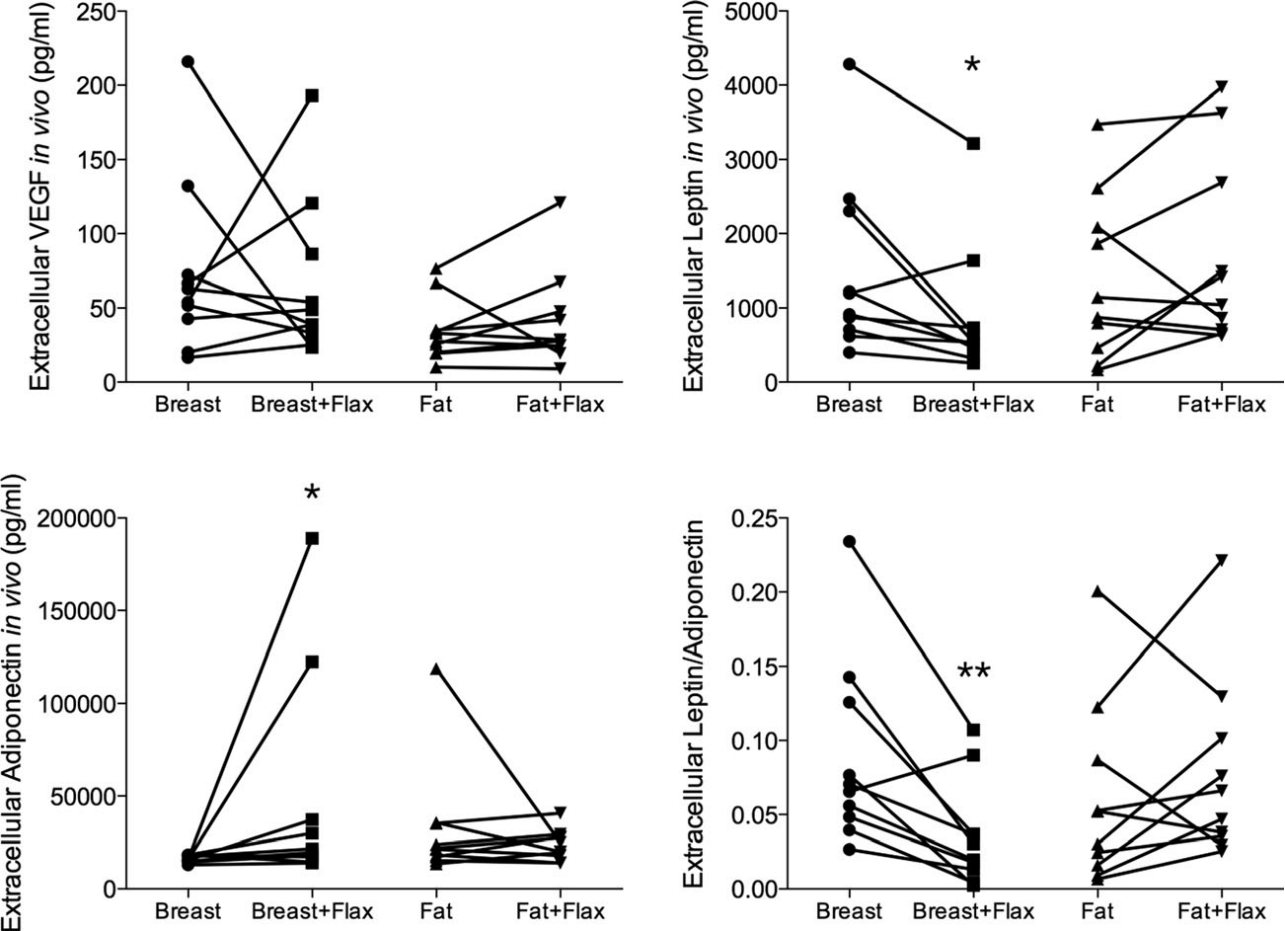
Dietary addition of flaxseed decreased leptin and leptin:adiponectin ratio in normal human breast tissue in vivo. Microdialysis of normal human breast tissue and abdominal subcutaneous (s.c.) fat was performed in two consecutive luteal phases in 10 premenopausal women before and after the addition of 25 g of ground flaxseed/day to their diet. *p < 0.05, **p < 0.01, *n* = 10

Microdialysis was performed in ten premenopausal women in two consecutive luteal phases of two menstrual cycles before and after the addition of 25 g of ground flaxseed to the diet for one month. No significant changes were detected in VEGF levels in breast tissue or s.c. abdominal fat tissue. In normal breast tissue the extracellular leptin levels decreased significantly and adiponectin levels increased. The ratio leptin/adiponectin was also significantly decreased in the breast. No significant changes were observed in abdominal s.c. fat tissue, Fig 4.

## Discussion

In this study, we show that VEGF correlated significantly with leptin and with the leptin:adiponectin ratio in normal human breast tissue *in situ*. No correlations were found in abdominal s.c. fat tissue. Co-culture of adipocytes and ER+ breast cancer cells induced alterations *per se* with increased extracellular levels of VEGF and leptin, and enhanced the effects of estradiol compared with either cell type cultured alone. *In vitro*, VEGF had a regulatory effect on leptin and not vice versa. A dietary addition of ground flaxseed decreased the in vivo extracellular breast levels of leptin and leptin:adiponectin ratio whereas VEGF levels were unaltered.

The cellular composition of the breast provides a unique microenvironment, contributing to several steps in the carcinogenic process [28]. This environment consists of different cell types such as epithelial-, endothelial-, immune-, and stroma cells and adipocytes. All of these cell types contribute to biologically active secreted factors in the extracellular space. This microenvironment is difficult to reproduce in *in vitro* systems and commonly used techniques such as immunohistochemistry of tissues may not reflect the extracellular components. Microdialysis is a minimally invasive *in vivo* technique, which enables sampling of molecules from the extracellular space of many organs. This sampling will, however, not distinguish from which cell type the molecules originate. The microdialysis technique has been adapted to sample bioactive molecules present *in situ* in both normal and malignant human breast tissue [15–17, 20, 29, 30]. By using microdialysis, it has previously been shown that estradiol regulates VEGF in normal human breast tissue *in vivo* and in breast cancer models [16–19, 30, 31]. VEGF, being a potent angiogenic factor, plays a key role in normal vascular growth, alongside with its involvement in several pathological conditions such as cancer [9]. In addition to estrogen, VEGF expression may also be regulated by several other factors including hypoxia, cytokines, and growth factors [32, 33]. In mouse mammary cancer cells, it has been shown that leptin increased the expression of VEGF and its receptor VEGF-R2 [8] suggesting that VEGF may be regulated by leptin. Our present data indeed support an interaction between leptin and VEGF in normal human breast tissue *in situ* as extracellular VEGF correlated significantly with leptin. None of these correlations were detected in abdominal s.c. fat suggesting tissue specific events in the breast. In our *in vitro* neutralizations experiments, inhibition of VEGF decreased extracellular leptin levels in a dose-dependent fashion whereas inhibition of leptin had no influence on VEGF levels. In contrast to previous animal studies, our data does not support the concept that VEGF is under direct regulation of leptin in human tissues. Although co-culture of two different cell types may not reflect the complex environment in the breast tissue, it offers a more physiologically relevant experimental model compared to single cell cultures. In our present study, co-culture per se increased the extracellular levels of leptin and VEGF compared with the different cell types cultured alone, which imply the importance of intercellular communication. Moreover, the effects of estradiol exposure seemed to be enhanced when the cells were co-cultured compared to monoculture. Co-culture of adipocytes and breast cancer cells resulted in a doubling of the leptin levels compared to adipocytes cultured alone whereas the levels were tripled in presence of estradiol. This highlights the importance of the cross talk between different cell-types for the biological function of a tissue. As all established so-called normal breast epithelial cell lines do not express the ER, we chose to use ER positive breast cancer cells to be able to elucidate the role of estrogen in these regulations [26, 27].

Life style factors such as diet have been shown to attenuate the risk of breast cancer in different populations [34, 35] and previous studies have shown that a dietary addition of flaxseed to premenopausal women tipped the breast microenvironment into angiogenesis inhibition and anti-inflammatory conditions [15, 21]. Here we show that flaxseed affected leptin and adiponectin levels whereas VEGF levels were unaltered. Adiponectin levels increased in eight out of ten women and in two women the increase was nearly ten-fold. The duration of the diet addition of flaxseed ranged between 26 and 33 days depending on the individual differences in menstrual cycle length. The ten-fold increase in the two women may reflect an altered response to the diet rather than a longer duration of the flaxseed addition as these women added flaxseed for 29 and 30 days respectively. Although our in vitro data suggested a VEGF dependent alteration of leptin the diet modification showed that leptin levels were affected even in absence of an alteration of VEGF. This suggests that flaxseed may regulate leptin and adiponectin by direct mechanisms or that indirectly mechanisms other than VEGF may be involved. Alterations of the microenvironment into a pro-tumorigenic state are key events in the initiation, growth, and progression of cancers including breast cancer. Autopsy studies have revealed that up to 15 % of all women have *in situ* cancer in their breast whereas only 1 % of women in the same age range are diagnosed with breast cancer [36]. A pro-tumorigenic environment may allow these *in situ* cancers to develop into clinical cancer disease. Our present data suggest that the breast microenvironment may be affected by relatively modest dietary modification and that flaxseed may induce an anti-tumorigenic microenvironment.

In summary, this study shows that extracellular VEGF exhibits a positive correlation with leptin in normal human breast tissue in vivo. The *in vitro* cell culture experiments imply that VEGF affects leptin expression and not vice versa. Co-culture with ER+ breast cancer cells and adipocytes induced profound changes of the extracellular environment *per se* and enhanced the effects of estradiol. Dietary addition of flaxseed resulted in decreased leptin and increased adiponectin levels in normal human breast tissue. There is a need to develop safer and less toxic breast cancer preventive strategies. Diet alterations could be one preventive breast cancer measure and the regulation of the breast microenvironment needs to be taken into account in future mechanistic studies of such interventions.
